# Measurement of Water Level in Urban Streams under Bad Weather Conditions

**DOI:** 10.3390/s21217157

**Published:** 2021-10-28

**Authors:** Joaquim Amândio Azevedo, João André Brás

**Affiliations:** Faculty of Exact Sciences and Engineering, University of Madeira, 9020-105 Funchal, Portugal; jbras1998@hotmail.com

**Keywords:** urban monitoring, water stream channels, water level measurement, image processing

## Abstract

Flood control and water resources management require monitoring the water level in rivers and streams. Water level measurement techniques increasingly consider image processing procedures. Most of the systems use a staff gauge to support the waterline detection. However, these techniques can fail when applied to urban stream channels due to water undulation, debris on the water surface, and traces of rain captured by the camera, and other adverse effects on images can be quite dramatic on the results. The importance of considering these effects is that they are usually associated with the variation in the water level with the occurrence of rain. The technique proposed in this work uses a larger detection zone to minimize the effects that tend to obstruct the waterline. The developed system uses an infrared camera to operate during the day and night. Images acquired in different weather conditions helped to evaluate the proposed technique. The water level measurement accuracy was about 1.8 cm for images taken during the day and 2.8 cm for images taken at night. During short periods of heavy rain, the accuracy was 2.6 cm for the daytime and 3.4 cm for the nighttime. Infrared lighting can improve detection accuracy at night. The developed technique provides good accuracy under different weather conditions by combining information from various detection positions to deal with waterline detection issues.

## 1. Introduction

Monitoring the water level in rivers, streams, and reservoirs has several applications, such as flood control, water flow measurement, and water resources management [1,2,3,4,5]. The techniques typically employed to measure the water level are based on float or pressure sensors, ultrasonic water meters, satellite-based systems, and image-based systems [6]. Owing to the risk of flooding, it is essential to control the flow of urban streams in hydrographic basins with a large population close to mountainous areas. In heavy rainfall situations, the water can drag large amounts of sediment and organic matter, rendering contact measurement systems unusable. Ultrasonic systems are simple to install, but they have several disadvantages, namely those related to water turbulence [7,8]. Satellite systems do not provide sufficient spatial or temporal resolution, especially for small water streams [9,10]. Image-based systems are a viable alternative to measure water level due to their low cost and easy installation beside a river or near houses [11,12,13]. Image analysis techniques to estimate the water level seem to be the most suitable for urban stream channels. While some works propose unmanned aerial vehicles with image acquisition capabilities for water monitoring [14,15,16], a fixed system is best suited to the context of this work.

Numerous works propose extraction of the waterline in image-based systems using a staff gauge to support the measurements. Hies et al. [17] applied an edge detection algorithm and the Hough transform [18] to detect the waterline over a white ruler located in the wall of an urban stream channel. Lo et al. [4] used images captured every minute to monitor the water level in urban riverine areas. They discarded images with low contrast or low brightness from the analysis. A water ruler on a bridge pier made it possible to monitor the water level. The authors also proposed the use of virtual markers when there is no ruler in the monitored zone. Lin et al. [19] determined an average image from successive images to reduce noise and applied the Hough transform to identify the waterline. They reported an accuracy of 1 cm when using single-camera images. Zhang et al. [20] proposed a system based on an infrared video camera to solve problems of poor visibility, image distortions, and ambient noise in water level measurements with staff gauges. A photovoltaic system of 200 W and a wind generator of 300 W supported by 12 V @300 Ah batteries provided the power to the camera and communication system. The method proposed in [21] deals with different illumination conditions of the water gauge. The authors used the difference between two adjacent regions of interest in the gray image, first with coarse regions to detect a zone for the waterline and then with fine positioning of the waterline. Xu et al. [22] proposed to improve the waterline detection accuracy by identifying the characters on the staff gauge image through a neural network. Image recognition with a staff gauge is also used in [23,24], obtaining a measurement error of 0.9 cm.

Some image-based water level measurement systems do not use staff gauges. The land region of the stream channel may have some texture allowing for the discrimination of the water region [25]. Griesbaum et al. [26] extracted the waterline along a building facade during flood events. Ridolfi et al. [27] proposed a method to obtain the waterline in dam reservoirs, where the high contrast between the concrete face and the water helped the decision. They applied the Canny method [28] to detect the water level. In the context of mountain streams, Young et al. [29] used several vertical rocks where a clear edge allowed the definition of the water margin. They manually removed images without a clear edge at the water margin from the detection process. Leduc et al. [30] considered a different method to obtain the water level of a mountain river, but they also removed images taken under bad weather conditions, like those obtained during rainfall events. Eltner et al. [31] deployed ground control points to provide a reference for the image data. To obtain the waterline, they used time-lapse images to highlight the water regions due to moving water.

Most of the techniques proposed in the literature to measure the water level in rivers and streams use staff gauges. The methods employed can be edge detection of the waterline, image thresholding to recognize the water surface, or character recognition on the staff gauge. Some techniques have used successive frames of images to improve waterline detection. Nevertheless, the presence of debris in the water obstructing the staff gauge and insufficient illumination make the process of water-level measurement difficult. In many cases, existing techniques discard images with low contrast or insufficient brightness. However, these images can be captured at night or during periods of rain in situations where water level measurement is more important. Several studies show high accuracy in detecting the water level, some of them providing results around 1 cm. While this is true for cases where the water has almost no undulation, other situations may impose lower accuracies owing to water level fluctuations. For instance, the work presented in [26] indicated an accuracy of 5 cm for water undulation of ±10 cm. This issue is rather important in periods of heavy precipitation. It should also be considered that raindrops on the camera lens could affect images because of outdoor installation.

In this work, we proposed a technique based on an image system to measure the water level in urban stream channels. Walls often flank these narrow-width streams. Heavy rainfall occurring in the surrounding mountains can cause rapid changes in the water level. In these situations, the water can drag large amounts of sediment and organic matter. These occurrences change the water quality and floating debris, making it difficult to use existing techniques to measure the water level. Therefore, we developed a new technique to deal with the debris on the water or obstacles in the waterline and consider different weather conditions. The technique does not require a staff gauge. A new approach became necessary to relate the image plane to the object plane that simplifies the parameterization needed to measure the water level. The image acquisition system is of low cost, autonomous in energy supply, and it makes use of the easy access to communication facilities normally found in urban areas. It also enables local processing to launch alerts, if desired, and internet access to send images to a remote server.

## 2. Materials and Methods

### 2.1. Measurement System

Flash floods have given rise to the greatest natural disasters on Madeira Island, with significant loss of human life. Given the orography of the island, with the highest point at 1862 m, heavy rains have caused strong water flows in the streams of the city of Funchal. From the beginning of the 19th century to the end of 2010, 38 flash floods were recorded on Madeira Island [32]. About 1000 people died in the flash flood of 1803, mostly in Funchal. More recently, the flash flood of 20 February 2010 resulted in more than 45 deaths. The weather station near Funchal recorded an accumulated rainfall above 4000 mm between October 2009 and February 2010, with some days recording a precipitation above 100 mm [33].

**Figure 1:**
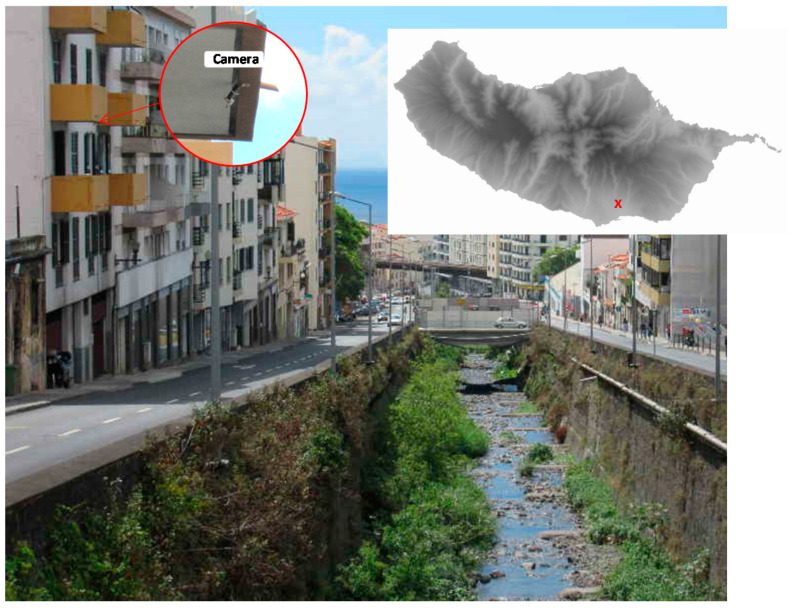
Urban stream channel image of the experimental setup.

Figure 1 shows an image of the channel used in the experimental setup to support the development of the proposed technique. This is one of the three main water streams of Funchal with a high potential risk of flooding. The figure also illustrates the region of Madeira where the study took place. Stone or concrete walls typically flank these urban streams. The installation of a staff gauge to provide a reference system for the waterline detection proved difficult or impossible due to the water flow being too strong during heavy rain events. Thus, this created a reference system obtained from natural existing control points on the channel wall.

We developed a low-cost image acquisition system based on a Raspberry Pi 3 model B and a Pi NoIR camera V1 [34]. This infrared camera allows for daytime as well as nighttime operation with different luminosity conditions. The camera was installed on the ceiling of a balcony in a building facing the stream. This method of installing the camera has several advantages. As the camera is under a balcony, and therefore protected from the rain, the lens becomes sheltered from raindrops. This installation also protects the camera from direct sunlight, which would saturate the image, and eliminates the need for a mast to suspend the camera, minimizing the environmental impact. The system used the Wi-Fi network of the house, avoiding the installation of a dedicated communication system. In the power supply for the camera, options were not limited to the house’s electrical system but also to a renewable energy system. For this study, we installed an 80 W solar panel on the building terrace for power supply and a 100 Ah @12V battery for energy storage. This solution makes the system autonomous in terms of power consumption.

### 2.2. Camera Calibration

The main parameters specified by the manufacturer for the Pi NoIR camera V1 are the resolution of 2592 × 1944 pixels, the pixel size of 1.4 μm × 1.4 μm, and the focal length of 3.6 mm. For camera calibration, we evaluated the focal length from experimental data. The dimension of an object in a plane parallel to the camera plane was given by

(1) $$w=\frac{P\times T\times d}{f}=\frac{\omega_{s}\times d}{f}$$

where *P* is the dimension of the object in pixels, *T* is the pixel size in mm, *d* is the distance between the camera lens and the plane of the object in meters, *w* is a dimension of the object in meters, *w_s_* is the image dimension of the object in mm, and *f* is the focal length in mm. We determined the focal length considering (1) with known distances, which yielded a result very close to the value provided by the manufacturer.

**Figure 2:**
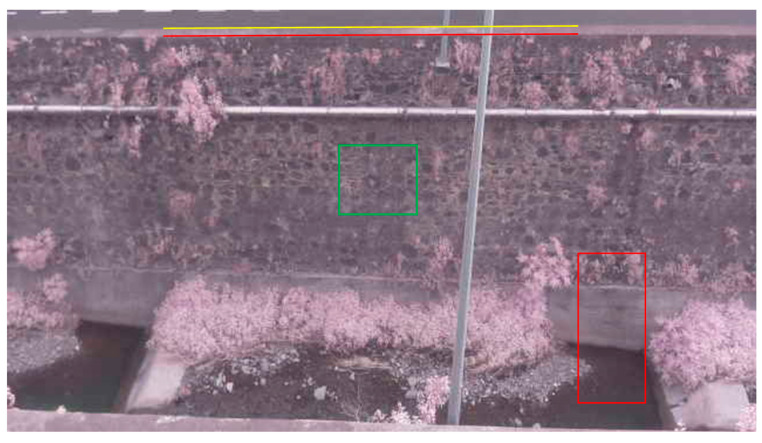
Image taken by the camera with the used control points (lines and green rectangle) and the ROI (red rectangle).

We found it necessary to determine some local parameters to define the region of interest (ROI) used to measure the water level. Figure 2 shows an image taken by the camera, with a resolution of 1280 × 720. In Pi camera V1, the change in the aspect ratio to achieve this resolution corresponds to 75% of the full sensor size in the vertical dimension. For data processing, the image should include the entire vertical region of the wall and part of the water zone. From initial experiments, we found that the wall was nine meters high and had an inclination of 5.8° related to the vertical. As the water stream is between two streets and for privacy reasons, an additional 15% cut to the top of the images became necessary to avoid capturing vehicles. Then, the images were converted to the resolution of 1280 × 720. The region of interest used in the water level measurements is marked in Figure 2 by a red rectangle. The figure also includes the control points used in this work, defined by the lines and the green rectangle. The region of interest included all vertical zones of the concrete face. From measurements, we determined that the concrete was two meters high.

**Figure 3:**
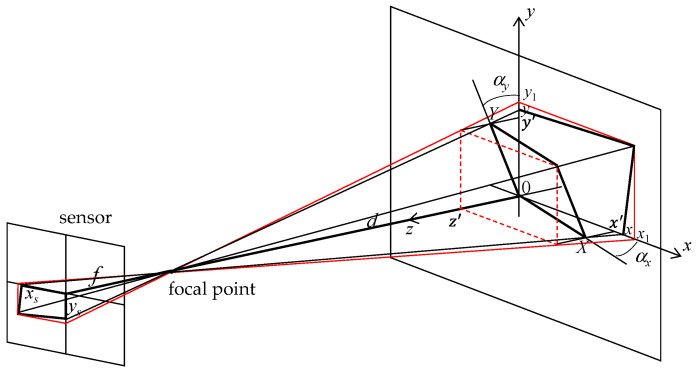
Reference system used to relate a point in the image plane to a point in the object plane.

Measurements proved to be a difficult task due to harsh access to the water stream. Thus, we determined the distance between the camera and the stream wall by an indirect method. Figure 3 shows the reference system used in the calculations. The plane *XY* is the object plane and *xy* is the image plane. The goal was to obtain the distance *d*, the horizontal angle *α_x_*, and the vertical angle *α_y_* to characterize the wall plane. These angles were defined in the *xz* and *yz* planes, respectively.

The horizontal and vertical distances were obtained from (1), giving

(2) $$x=x_{s}\frac{d}{f}\;\;\;\;\;\;\;\;\;\;\;\;\;\;\;\;y=y_{s}\frac{d}{f}\;\;\;\;\;\;\;\;\;\;\;\;\;\;\;\;\;$$

with

(3) $$x_{s}=P_{x}\times T\times \frac{S_{H}}{R_{H}}\;\;\;\;\;\;\;\;\;y_{s}=P_{y}\times T\times C_{1}\times C_{2}\times \frac{S_{V}}{R_{V}}\;\;\;\;\;\;\;\;\;\;\;\;\;\;$$

where *S_H_* is the horizontal sensor size (2592 pixels), *S_V_* is the vertical sensor size (1944 pixels), *R_H_* is the horizontal image resolution (1280 pixels), *R_V_* is the vertical image resolution (720 pixels), *C*_1_ is the image cut due to the aspect ratio change (0.75), and *C*_2_ is the second image cut (0.85). The development of a new approach proved necessary to relate the image plane to the object plane. The goal was to simplify the parametrization required to obtain the water level, given by the distance from the stream wall to the camera, the horizontal angle between both planes, and the vertical angle between both. Using the geometric representation of Figure 3 and considering *x*’/(*d*-*z*’) = *x*/*d*, the distance *X* can be obtained from *x* using the expression

(4) $$X=\frac{xd}{d\cos\left(\alpha_{x}\right)+x\sin\left(\alpha_{x}\right)}\;\;\;\;\;\;\;\;\;\;\;\;\;$$

Similarly, *Y* is given by

(5) $$Y=\frac{yd}{d\cos\left(\alpha_{y}\right)+y\sin\left(\alpha_{y}\right)}\;\;\;\;\;\;\;\;\;\;\;\;\;$$

For calibration purposes when performing actual measurements in the ROI, we placed a ten-meter graduated strip vertically on the wall in different horizontal positions. These measurements also provided values to assess the error made by the proposed technique in measuring the water level. From two distances obtained in the image in opposite directions around the origin, two vertical values *y_a_* and *y_b_* were defined using (2). The graduated strip made it possible to obtain the corresponding actual distances *Y_a_* and *Y_b_*. With (5) and these two distances, the unknowns *d* and *α_y_* could be determined by solving a system of two equations. The results were *d* = 23.68 m and *α_y_* = 27.2°. Substituting *d* into (4), *α_x_* was determined from known values of *x* and *X*, giving 7.4°.

To relate a point in the *xy* plane with a point in the *XY* plane, we derived the equation of the *XY* plane using the general form *ax* + *by* + *cz* + *d* = 0. The constants *a*, *b*, *c*, and *d* were obtained using three points of the plane. The points were (0,0,0), (0,*y*’,*z*’), and (*x*’,0,*z*’), resulting in the following plane equation in the *xyz* reference system:

(6) $$z=\tan\left(\alpha_{x}\right)x+\tan\left(\alpha_{y}\right)y\;\;\;\;\;\;\;\;\;\;\;\;\;\;\;\;\;$$

For a point (*X*,*Y*) in the object plane, a point (*x*_1_,*y*_1_) in the image plane is given by

(7) $$x_{1}=\frac{Xd\cos\left(\alpha_{x}\right)}{d-X\sin\left(\alpha_{x}\right)-Y\sin\left(\alpha_{y}\right)}\;\;\;\;\;\;\;\;\;\;\;\;\;\;\;\;\;$$

(8) $$y_{1}=\frac{Yd\cos\left(\alpha_{y}\right)}{d-X\sin\left(\alpha_{x}\right)-Y\sin\left(\alpha_{y}\right)}\;\;\;\;\;\;\;\;\;\;\;\;\;\;\;\;\;$$

A point in the object plane can be obtained from a point in the image plane by solving these equations for *X* and *Y*.

### 2.3. Camera Motion Compensation

**Figure 4:**
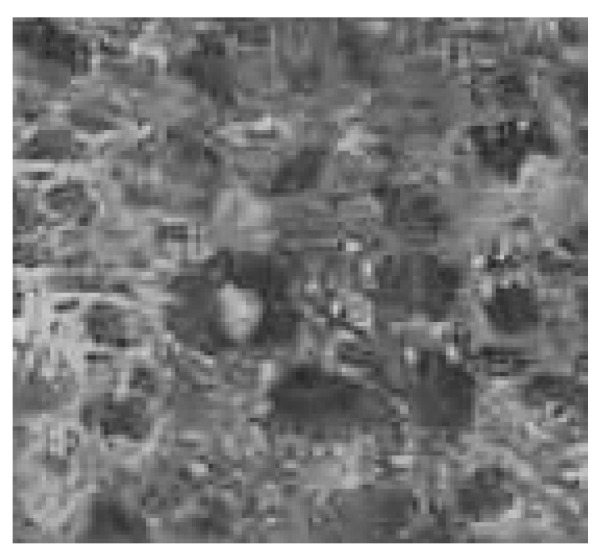
Template used in camera compensation.

Strong wind speeds can cause small camera movements. In addition, camera position may vary over time due to its reinstallation, which results in minor changes to the calibration parameters determined in the previous section. To obtain a stable ROI, it was necessary to calculate a camera motion compensation before any measurement of the water level. The motion compensation included image rotation and translation. We considered the line defined by the upper edge of the stream wall as a set of control points to determine the camera rotation about the initial conditions. Another control point was the coordinates of a template used to compensate for the translation motion. Figure 4 shows an example of a template applied in the compensation procedure. A green rectangle represents the template shown in Figure 2. Appropriate characteristics are necessary for the template to be detectable. To generalize the procedure proposed in this work, we removed the metallic tubes observed in Figure 2 from the template options. In any case, using this type of object increases the success rate of the template matching procedure.

**Figure 5:**
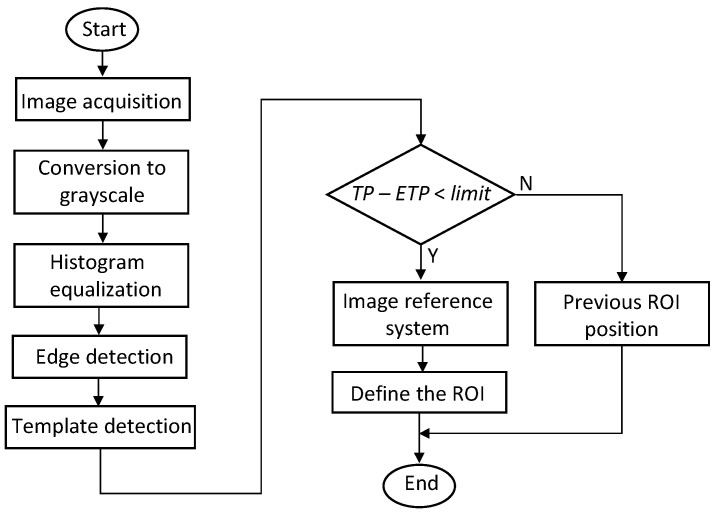
Flowchart for the camera movement compensation.

The flowchart shown in Figure 5 describes the camera motion compensation procedure. Python software allowed for data processing with the support of the OpenCV library [35]. The images acquired by the camera were converted to grayscale. We applied the Contrast Limited Adaptive Histogram Equalization (CLAHE) method [36] to highlight the wall features. The edge detection procedure started by applying a Gaussian filter to reduce noise and applying the binarization process to convert the grayscale image into a binary image. We applied this procedure to the area around the top of the wall containing the desired edges. For edge detection, we chose the Canny method because it provides the best performance among all edge detectors [37]. The Hough transform proved to be the most effective to identify the straight lines of the edges within the area of interest.

The edge detection procedure aims to detect the yellow or red line represented in Figure 2. In many situations, it detected both edges. In this case, the compensation procedure considered was the upper edge. In some cases, such as at night, this edge was not detected, and the second edge was necessarily used. As the two lines were parallel, the image rotation due to the camera motion was determined. Next, we applied a template matching method to detect the coordinates of the image given in Figure 4. This procedure allowed us to obtain the translation of the image caused by the camera movement. Six matching methods are available in the OpenCV library to search the template in the input image. The best results were obtained with the Normalized Correlation Coefficient Matching method. Finally, the coordinates of the template allowed for defining the ROI in the input image.

In most cases, the algorithm detected the correct wall edge and the correct template position. However, for images taken under very difficult lighting conditions, incorrect detection of one of these parameters or both may occur. In this case, the error made in detecting the waterline when using an incorrect ROI setting can be high. To avoid this situation, we determined the distance between the edge and the position of the template (TP) and compared it with the expected position of the template (ETP). When this difference exceeded a certain limit, we applied the last successful compensation to the ROI. As the algorithm does not know which edge it detects, the comparison represented in the flowchart served for the two values of the *limit* parameter. We determined this parameter by measuring the distance between the edge and the position of the template for various images acquired in different situations. It may also happen that the algorithm detects the edge with a small error in the slope and the template position correctly. This situation can lead to errors in setting the ROI. The application of a second template proved to be useful to minimize this effect.

### 2.4. Waterline Detection

**Figure 6:**
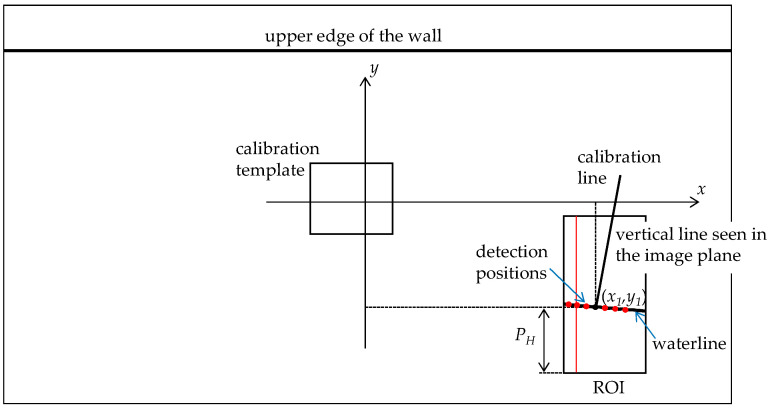
Schematic of the image plane.

The waterline detection procedure started by defining a ROI around the water boundary, as shown in Figure 2. We set this region at a certain distance from the center of the image. However, small camera movements can result in an image center different from that obtained in the calibration process, which can cause large errors in the waterline detection. Another way was to define the ROI using a reference point of the stream wall around the center. This point can be the coordinates of the template used for camera compensation. Figure 6 shows the image reference system defined to support the waterline detection. A vertical line in the object plane is seen in perspective in the image plane. We used the line within the ROI to detect the waterline position, defined by the point (*x*_1_,*y*_1_).

**Figure 7:**
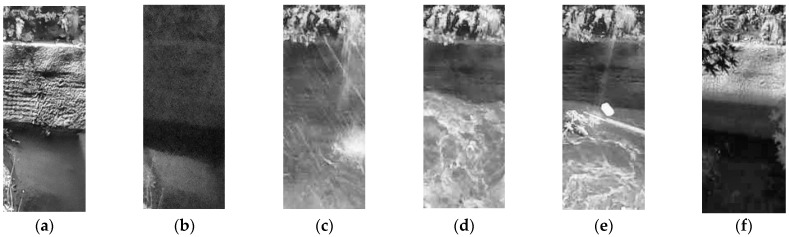
Different conditions of the waterline detection: (**a**) taken during the day; (**b**) taken at night; (**c**) image with traces of rain; (**d**) water undulation; (**e**) debris in the water; (**f**) shaded zone on the ROI.

Figure 7 shows the ROI for various images taken under different conditions. The stream flow can be characterized by shallow water most of the time, resulting in images like the one shown in Figure 7a taken during the day. Figure 7b shows a typical image taken at night. Rain events affect image quality, as shown in Figure 7c. Figure 7d is a typical situation that occurs during periods of rain, with water undulation. Another situation is the existence of debris on the water surface, as shown in Figure 7e. Figure 7f shows an example with a shadow effect within the ROI created by buildings on sunny days. As can be seen, edge detection methods are not suitable for obtaining the waterline because of the image and water quality.

To minimize some of the effects observed in the images of Figure 7, we considered *T* images captured with a time difference of three seconds between them to obtain an average image. By converting the image to grayscale and applying histogram equalization, it became possible to highlight the waterline. This line was determined by detecting the transition between the water and the stream wall. We also defined a reference system for the ROI to support the waterline detection procedure, where (*x*’,*y*’) is a point in the ROI image with the origin at the lower-left pixel. The application of a moving average filter allowed reducing noise effects on the image. For a position *x*’, represented in Figure 6 by a red line, the grayscale profile was given by

(9) $$P_{1}\left(x^{\prime }+\frac{N_{x}-1}{2},y^{\prime }+\frac{N_{y}-1}{2}\right)=\frac{1}{N_{x}N_{y}}\;\overset{N_{x}}{\underset{n=1}{\sum }}\overset{N_{y}}{\underset{m=1}{\sum }}P\left(x^{\prime },y^{\prime }\right),\;1\leq y^{\prime }\leq P_{y}-N_{y}\;\;\;\;\;\;\;\;\;\;\;\;\;$$

where *P*(*x*’,*y*’) is the pixel at position (*x*’,*y*’), *N_x_* is the number of horizontal pixels, *N_y_* is the number of vertical pixels, and *P_y_* is the number of pixels in the vertical dimension of the ROI. In addition, we determined the gradient of the grayscale profile to detect the water boundary by the maximum absolute value of the gradient.

**Figure 8:**
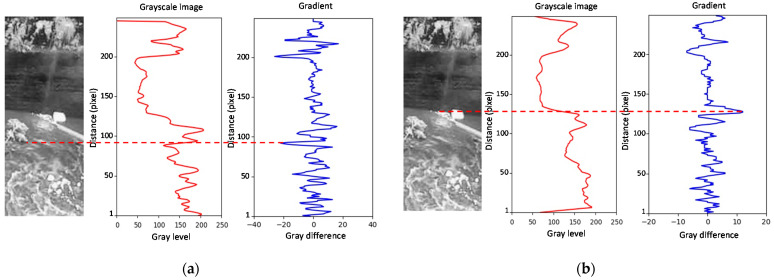
Waterline detection for an image with debris in the water: (**a**) *S* = 1; (**b**) *S* = 10.

The problem of using a single detection position was that, in many situations, the maximum absolute value of the gradient did not match the position of the waterline. Irregularities in the wall, debris on the water surface, traces of rain captured by the camera, water undulation, and other effects can create a maximum gradient at the wrong position. Using a larger waterline zone solved this problem and improved detection. For the experimental setup, we surveyed the waterline at *S* equidistant positions (detection positions) in a dimension of about two meters. We added the gradients of the grayscale profiles considering the slope of the waterline to enhance its detection (Figure 6). For this, we measured the slope *m* of this line, giving a relationship between *y*’ and *x*’ of the form Δ*y*’ = *m* Δ*x*’. The gradients of the grayscale profiles were determined at *S* positions of *x*’. The sum of gradients considered the slope of *y*’ to highlight the waterline values and to minimize the effects that degrade the waterline detection. In other urban stream locations, the waterline inside the ROI may have a different shape. The applied procedure uses the detection positions defined on the curve created by the waterline. Figure 8 shows the result obtained with one detection position (Figure 8a) and ten detection positions (Figure 8b) for an image with debris on the water surface. For *S* = 1, the maximum absolute value of the gradient was drastically affected by the water quality. As shown in Figure 8b, it was possible to detect the waterline with several detection positions, despite the existence of floating debris around the sensing zone.

**Figure 9:**
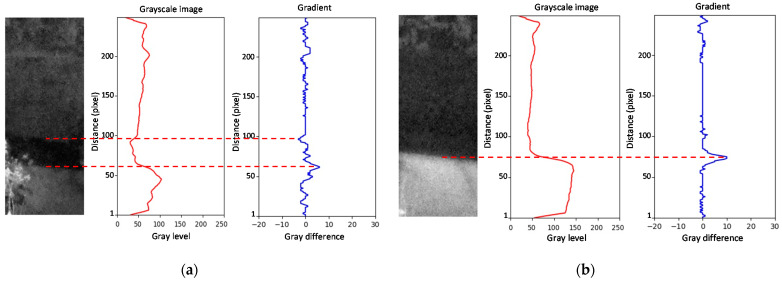
Waterline detection image taken at night: (**a**) the waterline is visible; (**b**) the waterline is invisible.

For images taken at night, it became necessary to consider the ROI lighting issues. As the water stream is in an urban environment, the street lighting system may be sufficient to illuminate the area of interest. In other cases, we can employ infrared lighting. In this work, we did not install any equipment to light the water zone, minimizing the costs of installing a dedicated system that requires a power supply. With the streetlights facing to the street, shadow zones might be visible within the ROI created by the stream wall. Thus, it was necessary to distinguish the procedure for obtaining the waterline for images taken at night from those taken during the day. The knowledge that night image acquisition requires different camera parameters allowed us to distinguish the two cases. Figure 9 shows the grayscale profile and the gradient for two images taken at night. Figure 9a illustrates an image with a detectable waterline. As can be observed, the maximum absolute value of the gradient occurred on the line created by the wall shadow over the water and not on the waterline. However, this position can aid in the detection of the water level. A variation in the water level has a corresponding variation in the shadow edge. The distance between the waterline and the shadow line was practically constant and defined by the parameter *P_W_* measured in the vertical of the image. Figure 9b shows a case where the waterline was not detected, and the shadow line was necessary to detect the water boundary.

**Figure 10:**
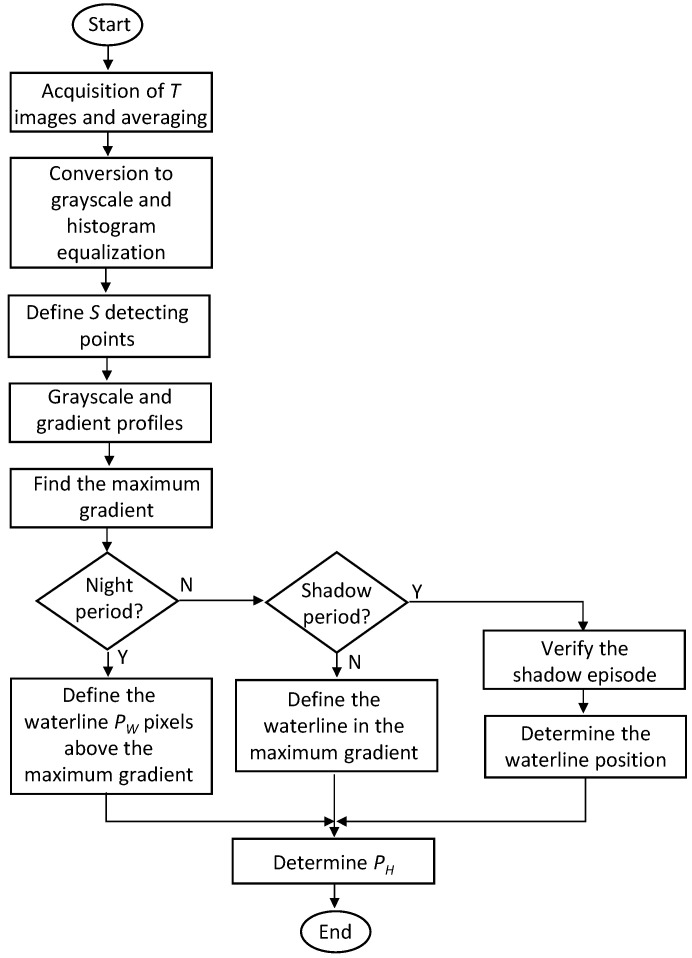
Flowchart for waterline detection.

Figure 10 shows the flowchart of the procedure to extract the waterline. The initial operations were the acquisition of *T* images to obtain the average image, conversion to grayscale, and histogram equalization. We defined *S* positions in the horizontal dimension of the ROI to detect the waterline. The grayscale profile was determined using (9) for each of the *S* values of *x’* as well as the corresponding gradient functions. Then, we obtained the maximum absolute value by summing the gradients.

Two situations arose for images taken during the day. The first one was the case in which the maximum absolute value of the gradient corresponded to the waterline. This situation happened most of the time and the algorithm searched for this maximum in the concrete face. We determined the parameter *P_H_* (in pixels) shown in Figure 6 from this maximum. However, for a short period during the day, shadows of buildings may appear in the ROI. In this case, the maximum absolute value of the gradient can occur in the transition between the sunlit area and the shaded area. In the flowchart of Figure 10, the “Shadow Period” defines the time interval in which this situation can happen, and we determined this period from initials measurements. To determine the waterline position, the algorithm searched for two peaks corresponding to the highest absolute values of the gradient. To assess whether the shadow of buildings affected the waterline detection procedure, we employed a technique to verify the conditions of existence of a shadow episode within the ROI. As sunlight produces a bright image in the sunlit zone, we determined, by image processing, the brightness of a small band above each of the gradient peaks and the brightness of a band below those peaks. This operation allowed us to compare the brightness of the image produced in the sunlit area with that of the shaded area. If any peak produced a difference in brightness above a threshold, this corresponded to a shadow transition. In that case, the other peak defined the position of the waterline. The threshold obtained came from measurements made on several captured images. Otherwise, we defined the waterline by the maximum absolute value of the gradient.

### 2.5. Water Level Estimation

To estimate the water level, we resorted to using Equations (7) and (8) to obtain *X* and *Y*, with the parameters of the wall plane of the concrete zone. We needed three parameters for this procedure, the distance *d*, the horizontal angle *α_x_*, and the vertical angle *α_y_*. Through measurements and simulation, we confirmed that the plane of the concrete zone was different from the plane of the stone zone. Following the procedure applied in the calibration section, the concrete plane had the following parameters: *d* = 24.16 m, *α_x_* = 7.4°, and *α_y_* = 22.2°.

With the *P_H_* parameter determined in the previous section, we obtained the position of the waterline in the reference system represented in Figure 6. The vertical distance is *P_y_* = *P_H_* − *P_y_*_0_, where *P_y_*_0_ is the origin of the ROI in the vertical dimension of the image. *P_x_* was determined from the following expression:

(10) $$P_{y}=m_{l}P_{x}+K\;\;\;\;\;\;\;\;\;\;\;\;\;\;\;\;\;$$

where *m_l_* and *K* are the calibration line parameters. Knowing *P_x_* and *P_y_*, *x*_1_ and *y*_1_ were calculated, respectively. Considering Equations (7) and (8), the distances in the wall plane are given by

(11) $$X=\frac{dx_{1}}{x_{1}\sin\left(\alpha_{x}\right)+y_{1}\cos\left(\alpha_{x}\right)\tan\left(\alpha_{y}\right)+d\cos\left(\alpha_{x}\right)}\;\;\;\;\;\;\;\;\;\;\;\;\;\;\;\;\;$$

(12) $$Y=\frac{y_{1}X\cos\left(\alpha_{x}\right)}{x_{1}\cos\left(\alpha_{y}\right)}\;\;\;\;\;\;\;\;\;\;\;\;\;\;\;\;\;\;\;$$

Finally, we determined the water level through the difference between *Y* and *Y*_0_, where *Y*_0_ is the distance considered for the zero-water level.

For some images, it was very difficult or impossible to measure the water level. In such cases, large errors could occur. We used data filtering to minimize the error effects. One procedure was to remove values that exceed a certain threshold when compared to previous results. To support this decision, we noticed that water flow could increase suddenly but decreases more slowly. The second peak of the gradient can also be applied to replace the wrong value if it does not exceed the defined threshold. The reason was the high probability of the waterline being there.

## 3. Results and Discussion

Images captured over several months allowed us to evaluate the developed technique. The stream water had very low levels for long periods, especially in summer. There was a noticeable variation in the water level only during episodes of rainfall in the surrounding mountains.

**Figure 11:**
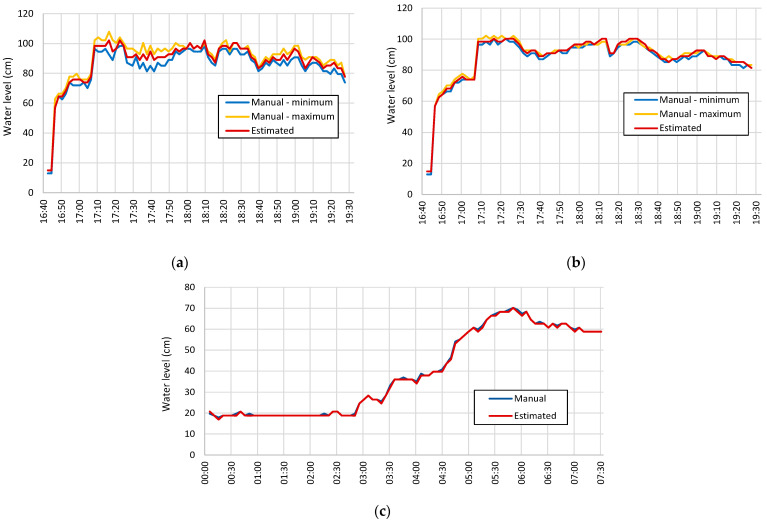
Water level estimation: (**a**) images taken during the day with *T* = 1; (**b**) image taken during the day with *T* = 5; (**c**) images taken at night.

Figure 11a,b show the results for images taken every two minutes on 3 April 2020. The rain event started during the day. The images were not affected by traces of rain because the precipitation occurred on the mountain. The camera motion compensation procedure allowed a correct configuration of the ROI, meaning a correct detection of the wall edge and the template. To evaluate the water undulation magnitude, Figure 11a shows the case for the water level measurement with a single image and *S* = 10 positions. Equation (9) defined *N_x_* = *N_y_* = 5. The graph shows the comparison between the values estimated by the proposed technique and the values measured manually from the images and the graduated strip. The average water undulation was 5.7 cm and the maximum undulation was 15.3 cm. The accuracy of the water level estimation was 0.9 cm. There was a lot of debris floating around the waterline between 5:30 pm and 7:10 pm. Temporal and spatial averages enabled us to minimize its effects in the estimation of the water level. Figure 11b shows the results with the average image determined from *T* = 5 images. The accuracy of the water level estimation was 0.8 cm for water undulation of 1.8 cm. This accuracy was possible because the technique combines information from different positions of the waterline to increase the success rate.

For the night period, we determined the parameter *P_W_* before estimating the water level. To obtain this parameter, we measured the shadow width for various water levels. The result was *P_W_* = 32 ± 2 pixels obtained with data from twenty cases. The accuracy of the water level estimation by this process was 2.2 cm. The technique applied to images acquired at night for the same day resulted in an accuracy of 2.6 cm. The error obtained for this period was higher because of the indirect estimation of the water level. Figure 11c shows the results for the case of images acquired every 5 min on 20 February 2021, with a rain event starting at night. The graph considers images acquired every 5 min. In this case, manual values correspond to results obtained by the shadow line. The accuracy of the water level estimation was 2.4 cm.

**Figure 12:**
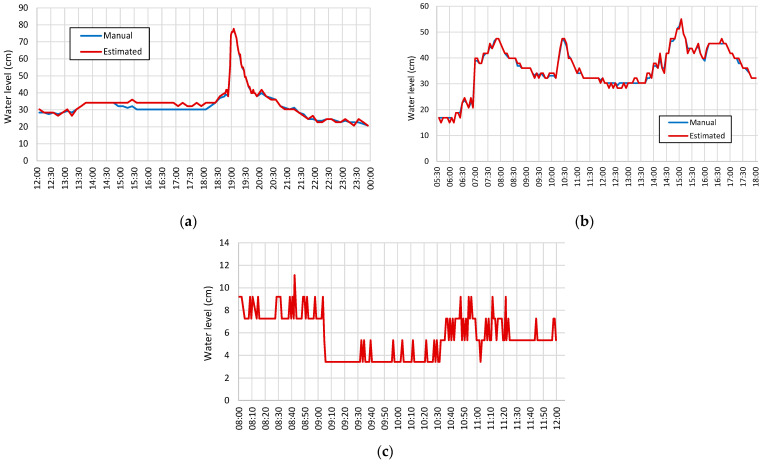
Water level estimation for images for different situations: (**a**) day with a change in the weather conditions; (**b**) day with heavy rain episodes; (**c**) shadow episode on the ROI.

Figure 12a shows the results obtained on 17 April 2020. The water underwent a small increase in level on a sunny day for the period between 1:20 pm and 1:40 pm and then slowly decreased. We may observe an error of 3 to 4 cm during this period because of the humidity on the wall, which makes the water level decision be slightly higher than the correct one. After 6:45 pm, the rain reached the urban area and the flow increased drastically. The accuracy of the estimate was 1.2 cm for the daytime and 2.8 cm for the nighttime. Another test was to evaluate the technique in periods with heavy rain, producing rain traces on acquired images. Figure 12b shows the results of an episode of this type on 27 March 2021. The accuracy had similar values to those obtained previously. Evaluating the results of two heavy rain events that occurred in December 2020 during the day, the accuracy was 1.8 cm. Rain events occurring at night produced an accuracy of 2.8 cm, being less affected than during the day as the image quality was more stable. Evaluating several heavy rain events, the accuracy obtained during short periods was about 2.6 cm for images taken during the day and 3.4 cm for images taken at night.

**Figure 13:**
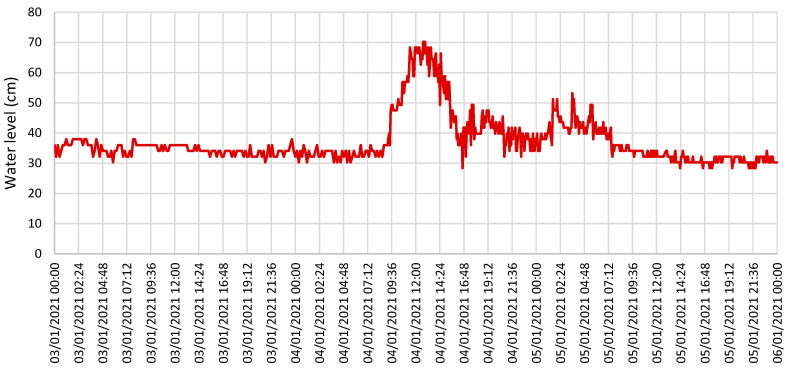
Water level estimation for a longer period of observation.

Another situation that required evaluation was the occurrence of shadows of buildings within the ROI. In the flowchart of Figure 10, we refer to this episode as the Shadow period. For the site of the experimental setup, this period occurred in the morning and lasted about 15 min. Considering data collected over a year, the determined shadow period was between 8:10 am and 9:10 am, because the shadow created by the buildings varied with the movement of the sun. It is also worth noting that the shadow episodes coincided with periods of shallow water. Figure 12c shows results for 10 June 2021 with a shadow episode occurring between 8:50 and 9:05 a.m. The water level was 5.3 cm in the time interval between 8:00 and 12:00. Before 9:05 a.m., larger errors occurred because the waterline area became too dark in the shaded zone. Due to the movement of the sun, different image qualities resulted in variations in the estimated water level. The accuracy obtained for these results was 1.6 cm. Figure 13 shows the results obtained for a longer period of observation. Images acquired between 3 and 5 January 2021 aided in estimating the water level. There was an episode of heavy rain starting at 9:00 a.m. on day 4 and some water level fluctuations during the night of day 5 originated by the rain fall in the surrounding mountains.

According to the results, the accuracy of the proposed method for obtaining the water level was 1.8 cm for daytime images and 2.8 for nighttime images. The accuracy at night was lower but varied less. Various methods show accuracies of about 1 cm [19,20,21,22,23,24]. However, these methods use a staff gauge to create a strong contrast between the water surface and the stream wall, as well as to serve as a reference for measurements. Furthermore, in some cases, they used dedicated lighting at nighttime. For the water stream in question, the use of a staff gauge turned out to be impossible. The luminosity provided by streetlights helped in the image acquisition at night, which reduced system installation costs. This solution produced a larger detection error when compared with the accuracy achieved with images taken during the day. However, improving accuracy is possible via the installation of infrared lighting on the wall in front of the ROI. This solution would remove the error produced by indirect detection. To summarize, Table 1 shows the accuracy obtained based on the experimental data for different water stream situations.

Some methods also remove images taken during adverse weather conditions, such as those with low contrast and brightness, and acquired in periods of heavy rain [4]. In the proposed technique, all taken images to detect the water level and filtering eliminated random errors. Even with low image contrast, the waterline remained detectable. An average water undulation of 5.7 cm and debris on the water surface resulted in an accuracy of 0.9 cm. Existing methods also do not allow measuring the water level properly in the presence of debris on the water around the staff gauge. The proposed technique considers several detection positions to overcome this problem. There were at least ten detection positions over a width of about two meters to estimate the waterline. This procedure is compatible with the use of staff gauges. Effectively, a way to improve accuracy in the presence of objects obstructing the waterline could be through multiple staff gauges with a specified distance between them. Afterwards, we can apply the proposed technique.

## 4. Conclusions

We developed a monitoring system to measure the water level in urban stream channels. Stone or concrete walls typically surround these channels to contain the water. The water is at a low level most of the time, but it can change quickly with rain events. These degrade water quality, cause water undulation, give rise to debris floating on the water surface, create traces of rain in the acquired images, and so on. The developed system used a Raspberry Pi and a Pi NoIR camera to operate day and night. Installation took place on a building facing the water stream wall. This installation allowed for attaining a low cost and low environmental impact solution for monitoring the water level. Placing the camera under the balcony avoided image saturation due to direct sunlight. The location also reduced the wind and rain effect on the camera. Wall features provided natural control points for camera calibration and reference for measurements. Compared with other methods, a larger detection zone allowed for minimizing the effects that make it difficult to detect the waterline. Using various image quality situations, we established the accuracy of the water level. We determined the accuracy from experimental data for different water stream situations influenced by weather conditions. Accuracies ranged from 0.8 cm to 2.6 cm for daytime and from 2.6 to 3.4 cm for nighttime. Average accuracies of 1.8 cm for the day and 2.8 cm for the night were determined by averaging the results obtained from various data periods. Although the results may be lower than those provided by some works, we did not use a staff gauge and the technique can be applied in periods where other methods tend to fail. Future work is needed to improve the water level detection in cases where a large area of the waterline remains obstructed by vegetation, mainly when it is possible by inspection to detect some points of the water boundary.

## Figures and Tables

**Table 1:** Accuracy as a function of the water stream situation.

	Situation	Accuracy (cm)
Daytime	Water undulation of 1.8 cm and debris in the water	0.8
	Water undulation of 5.7 cm and debris in the water	0.9
	Shallow water, sunny day	1.6
	Rain	1.3
	Heavy rain	2.6
	Average for the observation period	1.8
Nighttime	Water undulation of 1.8 cm and debris in the water	2.6
	Rain	2.7
	Heavy rain	3.4
	Average for the observation period	2.8

## Data Availability

Not applicable.

## References

[1] Chen Y., Chiu C. (2002). An efficient method of discharge measurement in tidal streams. J. Hydrol..

[2] Hapuarachchi H.A.P., Wang Q.J., Pagano T.C. (2011). A review of advances in flash flood forecasting. Hydrol. Process..

[3] Chen Y. (2013). Flood discharge measurement of a mountain river—Nanshih River in Taiwan. Hydrol. Earth Syst. Sci..

[4] Lo S., Wu J., Lin F., Hsu C. (2015). Visual Sensing for Urban Flood Monitoring. Sensors.

[5] Fujita I. (2017). Discharge Measurements of Snowmelt Flood by Space-Time Image Velocimetry during the Night Using Far-Infrared Camera. Water.

[6] Bradley A.A., Kruger A., Meselhe E.A., Muste M.V.I. (2002). Flow measurement in streams using video imagery. Water Resour. Res..

[7] Yorke T.H., Oberg K.A. (2002). Measuring river velocity and discharge with acoustic Doppler profilers. Flow Meas. Instrum..

[8] Yu J., Hahn H. (2010). Remote Detection and Monitoring of a Water Level Using Narrow Band Channel. J. Inf. Sci. Eng..

[9] Alsdorf D.E., Rodríguez E., Lettenmaier D.P. (2007). Measuring surface water from space. Rev. Geophys..

[10] Gleason C.J., Smith L.C., Finnegan D.C., LeWinter A.L., Pitcher L.H., Chu V.W. (2015). Technical Note: Semi-automated effective width extraction from time-lapse RGB imagery of a remote, braided Greenlandic river. Hydrol. Earth Syst. Sci..

[11] Tsubaki R., Fujita I., Tsutsumi S. (2011). Measurement of the flood discharge of a small-sized river using an existing digital video recording system. J. Hydro-Environ. Res..

[12] Yang H., Wang C., Yang J. (2014). Applying image recording and identification for measuring water stages to prevent flood hazards. Nat. Hazards.

[13] Zhen Z., Yang Z., Yuchou L., Youjie Y., Xurui L. IP camera-based LSPIV system for on-line monitoring of river flow. Proceedings of the 2017 IEEE 13th International Conference on Electronic Measurement & Instruments.

[14] Tauro F., Porfiri M., Grimaldi S. (2016). Surface flow measurements from drones. J. Hydrol..

[15] Langhammer J., Bernsteinová J., Mirijovský J. (2017). Building a High-Precision 2D Hydrodynamic Flood Model Using UAV Photogrammetry and Sensor Network Monitoring. Water.

[16] Bandini F., Jakobsen J., Olesen D., Reyna-Gutierrez J.A., Bauer-Gottwein P. (2017). Measuring water level in rivers and lakes from lightweight Unmanned Aerial Vehicles. J. Hydrol..

[17] Hies T., Babu P.S., Wang Y., Duester R., Eikaas H.S., Meng T.K. Enhanced water-level detection by image processing. Proceedings of the 10th International Conference on Hydroinformatics.

[18] Duda R.O., Hart P.E. (1972). Use of the Hough transformation to detect lines and curves in pictures. Commun. ACM.

[19] Lin Y., Lin Y., Han J. (2018). Automatic water-level detection using single-camera images with varied poses. Measurement.

[20] Zhang Z., Zhou Y., Liu H., Gao H. (2019). In-situ water level measurement using NIR-imaging video camera. Flow Meas. Instrum..

[21] Zhang Z., Zhou Y., Liu H., Zhang L., Wang H. (2019). Visual Measurement of Water Level under Complex Illumination Conditions. Sensors.

[22] Xu Z., Feng J., Zhang Z., Duan C. Water Level Estimation Based on Image of Staff Gauge in Smart City. Proceedings of the 2018 IEEE SmartWorld, Ubiquitous Intelligence & Computing, Advanced & Trusted Computing, Scalable Computing & Communications, Cloud & Big Data Computing, Internet of People and Smart City Innovations.

[23] Guo S., Zhang Y., Liu Y. (2020). A Water-Level Measurement Method Using Sparse Representation. Autom. Control Comput. Sci..

[24] Chen G., Bai K., Lin Z., Liao X., Liu S., Lin Z., Zhang Q., Jia X. (2021). Method on water level ruler reading recognition based on image processing. Signal Image Video Process..

[25] Udomsiri S., Iwahashi M. (2008). Design of FIR Filter for Water Level Detection. World Acad. Sci. Eng. Technol..

[26] Griesbaum L., Marx S., Höfle B. (2017). Direct local building inundation depth determination in 3-D point clouds generated from user-generated flood images. Nat. Hazards Earth Syst. Sci..

[27] Ridolfi E., Manciola P. (2018). Water Level Measurements from Drones: A Pilot Case Study at a Dam Site. Water.

[28] Canny J. (1987). A Computational Approach to Edge Detection. IEEE Trans. Pattern Anal. Mach. Intell..

[29] Young D.S., Hart J.K., Martinez K. (2015). Image analysis techniques to estimate river discharge using time-lapse cameras in remote locations. Comput. Geosci..

[30] Leduc P., Ashmore P., Sjogren D. (2018). Technical note: Stage and water width measurement of a mountain stream using a simple time-lapse camera. Hydrol. Earth Syst. Sci..

[31] Eltner A., Elias M., Sardemann H., Spieler D. (2018). Automatic Image-Based Water Stage Measurement for Long-Term Observations in Ungauged Catchments. Water Resour. Res..

[32] Quintal R. (1999). Aluviões da Madeira—Séculos XIX e XX. Territorium.

[33] Oliveira R.P., Almeida A.B., Sousa J., Pereira M.J., Portela M.M., Coutinho M.A., Ferreira R., Lopes S. Avaliação do Risco de Aluviões na Ilha da Madeira. Proceedings of the 10° Simpósio de Hidráulica e Recursos Hídricos dos Países de Língua Oficial Portuguesa (SILUSBA).

[34] Raspberry Pi. https://www.raspberrypi.org.

[35] Bradski G. (2000). The OpenCV library. Dobb’s J. Softw. Tools Prof. Program..

[36] Pizer M.S., Amburn E.P., Austin J.D., Cromartie R., Geselowitz A., Greer T., Romeny B.H., Zimmerman J.B., Zuiderveld K. (1987). Adaptive histogram equalization and its variations. Comput. Vis. Graph. Image Process..

[37] Batra B., Singh S., Sharma J., Arora S.M. (2016). Computational analysis of edge detection operators. Int. J. Appl. Res..

